# A UK-wide survey of speciality doctors and associate specialist (SAS) psychiatrists' psychotherapy case experience, barriers and benefits to professional development and clinical capabilities

**DOI:** 10.1192/bjo.2021.446

**Published:** 2021-06-18

**Authors:** Alina Vaida, Masud Awal

**Affiliations:** Birmingham and Solihull Mental Health NHS Foundation Trust

## Abstract

**Aims:**

Research suggests that seeing psychotherapy cases benefits psychiatric trainees’ professional development and clinical capabilities, however there is lack of such evidence for SAS psychiatrists, who require this experience for Certificate of Eligibility for Specialist Registration (CESR) applications.

Having provided frequently requested psychotherapy training support to our Trust's CESR training programme in Birmingham we aimed to study nationwide SAS psychiatrists’ psychotherapy case experience, professional benefits and barriers to access.

**Method:**

An online questionnaire was sent to UK-wide SAS Psychiatry doctors, with the support of the RCPsych Speciality Doctors and Associate Specialist Psychiatrists Committee (SASC), whilst being promoted on social media. It asked about psychotherapy-related experience, barriers and plans.

**Result:**

122 doctors completed the questionnaire, estimated to constitute approximately 8% (or more if considering all vacancies) of SAS psychiatry posts based on the RCPsych Census (2015), from across all UK nations and regions.

23% had gained experience in delivering psychotherapy (57% of whom confirmed CESR or training application plans), seeing cases mainly in CBT (52%) and psychodynamic psychotherapy (41%). Those who had delivered psychotherapy agreed or strongly agreed that it helped them become a better listener (82%), become more empathetic (75%), enjoy work more (71%), understand the unconscious communication better (82%), be more confident about referring for psychotherapy (82%) and overall be a better psychiatrist (86%).

44% planned to start a psychotherapy case but had not started, of whom only 22% had identified a supervisor and 15% identified a case. Only 11% felt confident they could get the psychotherapy training experiences they needed. Barriers reported included it not being part of their job plan (70%), time constraints (57%), difficulties in accessing psychotherapy supervision (61%), difficulties in identifying suitable cases (32%) and limited knowledge about psychotherapy (30%).

**Conclusion:**

Doctors who delivered psychotherapy reported benefits on many levels, making a strong case it develops their clinical capabilities, which may facilitate psychologically-informed care.

The results indicate interest in psychotherapy training outstripped available opportunity and support. Whilst some barriers mirrored those previously reported for trainees (difficulties accessing supervision and cases) others identified particularly related to SAS workload (not being part of their job plan and time constraints) and lack of support (with trainees prioritised). This may highlight a potential concern given the SAS Charter covers CESR-related support and advocates appropriate Supporting Professional Activities (SPA) time.

Trusts need to consider more actively supporting SAS psychotherapy training and including in job planning for those receiving, delivering and supporting these valued experiences.

